# Efficacy and safety of rituximab-based chemoimmunotherapy in adult patients with Burkitt lymphoma in Korea

**DOI:** 10.3389/fonc.2025.1614506

**Published:** 2025-07-09

**Authors:** Gi-June Min, Ka Young Kim, Tong Yoon Kim, Young-Woo Jeon, Byung-Su Kim, Seung-Ah Yahng, Ki-Seong Eom, Seok-Goo Cho

**Affiliations:** ^1^ Department of Hematology, Seoul St. Mary’s Hospital, College of Medicine, The Catholic University of Korea, Seoul, Republic of Korea; ^2^ Department of Hematology, Yeouido St. Mary’s Hospital, College of Medicine, The Catholic University of Korea, Seoul, Republic of Korea; ^3^ Department of Hematology, Eunpyeong St. Mary’s Hospital, College of Medicine, The Catholic University of Korea, Seoul, Republic of Korea; ^4^ Department of Hematology, Incheon St. Mary’s Hospital, College of Medicine, The Catholic University of Korea, Incheon, Republic of Korea

**Keywords:** Burkitt lymphoma, rituximab, prognosis, performance status, chemoimmunotherapy

## Abstract

**Background:**

Burkitt lymphoma (BL), a rare, aggressive *MYC*-driven B-cell non-Hodgkin lymphoma (NHL), has endemic, sporadic, and immunodeficiency-associated variants. In Asia, BL accounts for 1–2% of lymphomas, with limited data available on adult outcomes. Although potentially curable, BL is associated with poor outcomes with low-intensity chemotherapy owing to rapid proliferation and chemoresistance. Therefore, high-intensity regimens including R-hyperCVAD/MC (Course A of rituximab, cyclophosphamide, doxorubicin, vincristine, and dexamethasone; Course B of rituximab, methotrexate, and cytarabine) have been commonly used; however, no optimal strategy has been established.

**Methods:**

This retrospective study included 69 adult patients with BL (age >15 years) diagnosed between 2009 and 2023 using the WHO criteria. Most of the patients were administered R-hyperCVAD/MC, while rituximab plus cyclophosphamide, doxorubicin, vincristine, and prednisone (R-CHOP) was administered to older patients or those with poor-performance-status to mitigate toxicity.

**Results:**

The median age of the patients was 55 years; 39.1% of the patients had Eastern Cooperative Oncology Group Performance Status (ECOG-PS) of 2–4, 62.3% had >1 extranodal site, 71.0% had stage IV, and 13.0% had central nervous system involvement. Furthermore, 13 (18.8%) patients were reclassified as BL after immunoglobulin heavy‐chain (IGH)/*MYC* detection. Overall, 52 patients were administered R-hyperCVAD/MC exclusively, 5 switched from R-CHOP, and 4 patients were primarily treated with R-CHOP owing to intolerance. At a median follow-up of 66.9 months, 5-year overall survival (OS) and event-free survival (EFS) were 69.5 and 65.2%, respectively and higher early mortality was observed in older patients (median survival: 3.9 months). Poor OS was associated with B-symptoms (hazard ratio [HR] 3.89, *p* = 0.003) and age ≥ 60 years (HR 2.54, *p* = 0.034); while poor EFS was associated with ECOG-PS 2–4 (HR 2.72, *p* = 0.024).

**Conclusions:**

Our study revealed that R-hyperCVAD/MC was effective but associated with high early mortality in older patients. Risk-adapted regimens and prognostic factors including age, B-symptoms, and ECOG-PS are crucial for optimizing treatment.

## 1 Introduction

Burkitt lymphoma (BL) is a rare, highly aggressive, *MYC*-driven B-cell non-Hodgkin lymphoma (NHL) (1). Three clinically different epidemiological BL variants have been identified including endemic, sporadic, and immunodeficiency-associated BL, mainly differing in their geographic distribution, clinical presentations, and biological features (1). Most cases of adult BL in Asia are sporadic with low incidence, accounting for only 1–2% of all lymphomas (1). Moreover, BL represents approximately 30–50% of all pediatric lymphomas; however, it accounts for < 5% of adult lymphoma cases (2). Therefore, few studies have characterized adult BL in Asia, and data on long-term clinical outcomes or prognostic factors for Asian patients with BL are scarce (3–5). Although BL is considered curable, outcomes have been reported to be poor in cases with low-intensity chemotherapy, including a CHOP-based regimen (6–8) owing to the high proliferation rate of BL cells, facilitating the re-entry of the remaining viable cells into the cell cycle, resulting in chemoresistance (8). Several highly intensive therapeutic regimens have been developed for BL in adults, targeting both the systemic and central nervous system (CNS) (3, 9–13). However, no optimal frontline treatment strategy has been developed. Therefore, in this study, we aimed to assess the potential of the R-hyperCVAD/MC regimen (Course A of rituximab, cyclophosphamide, doxorubicin, vincristine, and dexamethasone; Course B of rituximab, methotrexate, and cytarabine) with improved efficacy and tolerable toxicity profiles owing to the addition of rituximab to the conventional hyperCVAD/MC regimen in BL treatment (12). Moreover, we aimed to assess the demographic and disease-related characteristics and their association with clinical outcomes in a retrospective cohort of Korean adults with BL.

## 2 Methods

### 2.1 Patient enrollment

We retrospectively analyzed data from (age ≥ 15 years) 69 adult patients with BL diagnosed between November 2009 and August 2023 using the fifth edition of the World Health Organization (WHO) classification (14). Two well-experienced pathologists independently reviewed and confirmed the BL diagnosis based on morphological characteristics, CD10, CD20, Bcl-2, Bcl-6, and Ki-67 expression detected by immunohistochemistry, chromosomal translocation of t (8, 14), and *c-myc* overexpression detected by fluorescence *in situ* hybridization. We excluded patients with negative *myc* rearrangement and no expression of BCL-6 and CD10, or < 80% Ki-67 expression. The disease stage of all enrolled patients were assessed using computed tomography (CT) of the neck, chest, abdomen, pelvis, and fluorine-18 fluorodeoxyglucose positron emission tomography-CT (18F-FDG PETCT). Moreover, the patients routinely underwent bone marrow (BM) biopsy at the time of diagnosis and cerebrospinal fluid analysis during diagnosis and treatment. The demographic information and laboratory data of patients with BL were collected from medical records. Patients were stratified using the international prognostic index (IPI) score system with the Ann-Arbor staging. The study protocol was approved by the Institutional Review Board (IRB) and Ethics Committee of the Catholic Medical Center, Republic of Korea (approval number: KC24RASI0525). This study was conducted in accordance with the tenets of the Declaration of Helsinki and the need for informed consent was waived by the IRB as the study did not adversely affect the rights or welfare of the participants owing to the minimal risk associated with this retrospective study.

### 2.2 Initial treatment strategies

The R-hyperCVAD/MC regimen consisting of two alternating courses of R-hyperCVAD (Course A; 375 mg/m^2^ rituximab on day 1 and 11, 300 mg/m^2^ hyper-fractionated cyclophosphamide every 12 h for 6 doses on day 1 to 3, 50 mg/m^2^ doxorubicin on day 4, 2 mg vincristine on day 4 and 11, and 40 mg dexamethasone daily on days 1 to 4 and day 11 to 14) and R-HDMTX/ARAC (Course B; 375 mg/m^2^ rituximab on day 2 and 8, 1000 mg/m^2^ methotrexate on day 1 and 3000 mg/m^2^ cytarabine every 12 h for 4 doses on day 2 to 3) was used to treat patients with BL (12). Moreover, 300 mg of allopurinol and hydration were administered to patients in course A to prevent tumor lysis syndrome (TLS). Additionally, a single 6-mg dose of rasburicase was routinely administered 6 h before initiation of course A chemotherapy to prevent TLS after approval and reimbursement of rasburicase treatment by the National Insurance System of Korea in 2018 (15). In course B, as methotrexate is primarily excreted via the kidneys, the blood concentration of methotrexate and creatinine were routinely monitored and dose-adjusted leucovorin rescue and aggressive hydration were used as prophylactic strategies, as previously described (16). All patients received prophylactic intrathecal chemotherapy—consisting of 12 mg methotrexate, 40 mg cytarabine, and 50 mg hydrocortisone—once during each of courses A and B.

### 2.3 Response evaluation

Due to BL’s aggressiveness, the interim response evaluation was performed after completion of 2–3 cycles (4 to 6 courses) of R-hyperCVAD/MC. Treatment response evaluation was conducted according to the WHO response criteria. Complete remission (CR) was defined as no evidence of residual disease, partial response (PR) was defined as at least 50% tumor mass reduction as compared to that at treatment onset, progressive disease (PD) was defined as at least a 25% increase in lesion size, and stable disease (SD) was defined as patient response not reaching PR or PD (17). If the interim response was SD or PD, we considered changing the salvage chemotherapy regimen. The standard protocol in our institute prescribes four cycles of the R-hyperCVAD/MC regimen (eight alternating courses); however, in patients achieving CR with decreased intensive chemotherapy tolerance, the treatment was halted at three cycles (six alternating courses). We employed the R-CHOP regimen (rituximab 375 mg/m^2^, cyclophosphamide 750 mg/m^2^, doxorubicin 50 mg/m^2^, and vincristine 1.4 mg/m^2^ on day 1, plus oral prednisolone 100 mg/m^2^ on days 1–5) as an alternative in patients over 60 years of age and/or those with an Eastern Cooperative Oncology Group Performance Status (ECOG-PS) of 2–4, who were highly motivated to undergo treatment but were considered unfit for the R-hyperCVAD/MC regimen due to its high treatment-related mortality. All treatment-related adverse events were assessed according to the National Cancer Institute Common Terminology Criteria for Adverse Events (NCI-CTCAE), version 5.0.

### 2.4 Salvage treatment strategies

For patients who failed to achieve at least a PR or showed evidence of PD during first-line treatment, the regimen was switched to salvage chemotherapy. The salvage regimens included DL-ICE (dexamethasone, L-asparaginase, ifosfamide, carboplatin, and etoposide), R-DHAP (rituximab, dexamethasone, high-dose cytarabine, and cisplatin), ESHAP (etoposide, methylprednisolone, high-dose cytarabine, and cisplatin), or IVAM (ifosfamide, etoposide, cytarabine, and methotrexate), all of which are commonly used to treat relapsed or refractory high-grade lymphoma (18). BL recurrence is typically reported within 1 year, and salvage treatment, including allogeneic hematopoietic stem cell transplantation (allo-HSCT), is usually unsuccessful (19). However, we considered allo-HSCT in fit patients achieving PR to CR during salvage chemotherapy for curative intent. Autologous transplantation was not considered in this study as relapsed/refractory BL is mostly chemo-resistant and has challenges associated with attaining long-term remission only by chemotherapeutic agents. Patients deciding to undergo allo-HSCT were administered reduced-intensity conditioning regimens consisting of 30 mg/m^2^/day fludarabine for 6 days, 70 mg/kg/day melphalan for 1 day, and 800 cGy of fractionated TBI for 2 days (20). Strategies of graft-versus-host disease and infection were also established based on previous reports (20).

### 2.5 Statistical analysis

Clinicopathologic characteristics were compared using chi-square test or Fisher’s exact test. Overall survival (OS) and event-free survival (EFS) were calculated from the date of diagnosis to the date of the last follow-up visit or death by any cause and from the date of treatment initiation to the date of recognized disease progression, fatal events by any cause, or the last follow-up visit, respectively. OS and EFS were estimated using the Kaplan–Meier method and compared using the log-rank test. Non-relapsed mortality (NRM) was defined as death due to PD or during treatment, considering patients with relapse or disease-unrelated deaths as competing risks. The cumulative incidence of relapse (CIR) was defined as disease relapse with NRM considered as a competing risk. CIR and NRM were measured by cumulative incidence estimation, and comparisons between the groups were based on Gray’s competing risk method. Survival rates were compared for statistical differences using a log-rank test, and *p* values < 0.05 were considered statistically significant. Two-tailed *p* values were used in the analysis. The Cox proportional regression model was used for multivariate analysis to calculate survival hazard ratios (HRs), and variables were selected based on previous studies on the known prognostic factors (3, 6, 10, 12). The Fine–Gray proportional hazard regression model was used to calculate the HRs for cumulative incidences. All statistical analyses were conducted using R-software (version 4.3.3, R Foundation for Statistical Computing, 2024).

## 3 Results

### 3.1 Patient characteristics

This study included 69 patients with BL treated at our hospital. Of these, 47 were males (68.1%) and 22 were females (31.9%), with a 2:1 male predominance and a median age of 55 years (range 15 – 81). ECOG-PS score of ≥ 2 at diagnosis was observed in 27 patients (39.1%) and 47 (68.1%) had elevated LDH. Moreover, 28 patients (40.6%) had B-symptoms at diagnosis. A total of 48 patients (71.0%) were classified as Ann-Arbor stage IV, and 43 patients (62.3%) had involvement of two or more extranodal sites. All patients with stage IV lymphoma had multiple intra-abdominal lymphadenopathies with 26 (37.7%) patients with BM involvement, 10 (14.5%) with Leukemic phase, and 9 (13.0%) with CNS involvement. Therefore, more than half of the patients (44/69, 63.7%) were categorized as high-intermediate to high-risk based on IPI risk classifications. Furthermore, 13 patients (18.8%) had their diagnosis changed from high-grade lymphoma to BL following detection of *IGH/MYC* translocations. The clinical characteristics of the enrolled patients are presented in **Table 1**.

**Table 1:** Characteristics of patients with Burkitt lymphoma (n = 69).

Characteristics	Values
Age, median (range)	55 (15–81)
Age ≥ 60 years	24 (34.8%)
Sex	
Male	47 (68.1%)
Female	22 (31.9%)
Lactate dehydrogenase, median (range)	1,084 (144–12,330)
Normal	22 (31.9%)
Elevated	47 (68.1%)
ECOG performance status	
0–1	42 (60.9%)
2	27 (39.1%)
Ann-Arbor stage	
I	4 (5.8%)
II	13 (18.8%)
III	3 (4.3%)
IV	49 (71.0%)
Extranodal involvement ≥ 2	43 (62.3%)
Leukemic phase^†^	10 (14.5%)
BM involvement	26 (37.7%)
CNS involvement	9 (13.0%)
B-symptoms^*^	28 (40.6%)
IPI risk classification	
Low	19 (27.5%)
Low-intermediate	6 (8.7%)
High-intermediate	17 (24.6%)
High	27 (39.1%)
Diagnostic change from B-cell HGL to BL	13 (18.8%)
Initial treatment regimen	
R-hyperCVAD/MC	52 (75.4%)
From R-hyperCVAD/MC to R-CHOP^**^	8 (11.6%)
From R-CHOP to R-hyperCVAD/MC^§^	5 (7.2%)
R-CHOP^‡^	4 (5.8%)
Initial treatment response	
CR	49 (71.0%)
PR (4→PD & 1→died)	5 (7.2%)
PD	8 (11.6%)
Not evaluated	7 (10.1%)
Consolidation	6/50

BL, Burkitt lymphoma; BM, bone marrow; CNS, central nervous system; CR, complete remission; ECOG, Eastern Cooperative Oncology Group; HGL, high-grade lymphoma; IPI, international prognostic index; PD, progressive disease; PR, partial remission.

†Leukemic phase is defined as ≥ 20% blast in bone marrow or peripheral blood.

*B-symptoms were defined by patients complaining of at least one or more of the symptoms including fevers, drenching night sweats, and losing > 10% of body weight over six months.

**These patients were administered R-hyperCVAD/MC treatment and achieved complete remission after two cycles but showed decreased performance status. Therefore, we decided to administer two additional cycles of R-CHOP to complete the treatment.

§These patients underwent 2 cycles of R-CHOP; thereafter, their treatment was changed to R-hyperCVAD/MC owing to delayed BL diagnosis.

‡These patients received only R-CHOP chemotherapy based on their clinicians’ assessment that their poor performance status or advanced age would render them unable to tolerate the intensity of R-hyperCVAD/MC.

### 3.2 Treatment and response

In total, 69 adult patients diagnosed with BL according to WHO criteria between 2009 and 2023 were stratified by age and ECOG-PS (**Supplementary Figure S1**). Among these 69 patients, 60 patients (87%) who were <60 years of age or had an ECOG-PS of <2 initiated treatment with the R-hyperCVAD/MC regimen; 52 patients (75.4%) completed the full course of R-hyperCVAD/MC without switching regimens. Notably, 5 of the 52 patients were initially referred with a diagnosis of diffuse large B-cell lymphoma but were reclassified as having BL upon further evaluation at our institution. Eight patients (11.6%) who achieved CR after two cycles (four courses) of R-hyperCVAD/MC were transitioned to two additional cycles of R-CHOP owing to declining performance status. All eight patients remained in long-term remission and were alive at the last follow-up. The remaining nine patients (13%) initiated treatment with R-CHOP. This group included individuals with confirmed BL who were ineligible for R-hyperCVAD/MC owing to age ≥ 60 years or ECOG-PS ≥ 2 and those initially diagnosed with high-grade B-cell lymphoma who were later confirmed to have BL (**Supplementary Figures S1**, **S2**). Two patients, aged 31 years and 42 years, respectively, were treated with R-CHOP based on the judgment of the clinicians owing to poor performance status caused by a massive tumor burden, rendering R-hyperCVAD/MC intolerable. Among these two younger but unfit patients, one died of septic shock after the first R-CHOP cycle (Patient 6; **Table 2**). Two additional older patients were later confirmed to have BL but were not switched to R-hyperCVAD/MC owing to advanced age and poor ECOG-PS: one progressed after two cycles of R-CHOP and died without receiving salvage therapy (Patient 12; **Table 2**), while the other achieved CR. In five patients, the initial diagnosis of high-grade B-cell lymphoma was later revised to BL owing to the delayed identification of *MYC* rearrangement or aberrant *MYC* expression, complicating timely diagnosis. As treatment could not be delayed owing to rapid disease progression, R-CHOP was promptly initiated in all five cases. Following confirmation of the BL diagnosis, the regimen was switched to R-hyperCVAD/MC, resulting in long-term remission in these patients.

**Table 2:** Detailed clinical characteristics and long-term outcomes of treatment failure or patients with relapse (n = 23).

No.	Sex/Age	Stage	ECOG	CNS inv.	Treatment Regimens	Response	EFS (mo.)	OS (mo.)	Final outcomes
During 1^st^ cycle of chemotherapy									
1	M/42	IV	1	No	R-hyperCVAD	N/A	1.0	1.0	Died d/t TLS
2	M/65	IV	2	No	R-hyperCVAD	N/A	1.2	1.2	Died d/t fungal pneumonia
3	M/52	IV	3	No	R-hyperCVAD	N/A	1.3	1.3	Died d/t RSV pneumonia
4	M/55	II bulky	2	No	R-hyperCVAD	N/A	1.4	1.4	Died d/t septic shock
5	F/66	IV	1	No	R-hyperCVAD	N/A	1.5	1.5	Died d/t septic shock
6	M/31	IV	2	No	R-CHOP	N/A	2.1	2.1	Died d/t septic shock
7	M/69	IV	2	No	R-hyperCVAD	N/A	2.5	2.5	Died d/t TLS
During 2^nd^ to 3^rd^ cycle of chemotherapy									
8	M/18	IV	3	No	R-hyperCVAD	PD	2.9	6.2	Died, salvage treatment (DL-ICE 1 cycle) stopped
9	F/81	IV	2	No	R-hyperCVAD	PD	3.1	3.1	Died without salvage treatment
10	M/78	IV	3	No	R-hyperCVAD	PD	3.6	3.6	Died without salvage treatment
11	M/24	IV	1	No	R-hyperCVAD	PD	3.8	7.3	Died d/t septic shock during salvage treatment (DL-ICE 2 cycles)
12	M/73	IV	3	No	R-CHOP	PD	3.8	3.8	Died without salvage treatment
13	F/55	IV	2	No	R-hyperCVAD	PR	3.9	3.9	Died, d/t fungal pneumonia
14	M/57	IV	3	Yes	R-hyperCVAD	PR→CNS Relapse	3.9	9.0	Died, hospice care without salvage
15	M/68	IV	1	Yes	R-hyperCVAD	PD	4.4	6.3	Died, hospice care without salvage
16	M/63	IV	2	No	R-hyperCVAD	PD	4.7	8.5	Died, salvage treatment (DL-ICE 2 cycles → PD → DHAP 1 cycle) stopped
17	M/66	IV	1	No	R-hyperCVAD	PD	5.3	12.3	Died d/t septic shock during salvage treatment (DL-ICE 3 cycles → PD → DHAP 2 cycles)
After 3^rd^ cycle of chemotherapy									
18	M/36	IV	1	No	R-hyperCVAD	CR→CNS Relapse	6.8	10.5	Died, hospice care without salvage
19	M/61	IV	2	No	R-hyperCVAD	PR →Relapse	7.3	8.3	Died, hospice care without salvage
20	M/19	IV	1	No	R-hyperCVAD	PR→CNS Relapse	8.6	47.3	Alive, CR after salvage treatment (WBRT + DL-ICE 4 cycles → CR → AlloHSCT → CR)
21	M/55	IV	2	No	R-hyperCVAD	PR →Relapse	9.5	35.0	Alive, CR after salvage treatment (DL-ICE 3 cycles → PD → ESHAP 4 cycles → PR → AlloHSCT → CR)
22	F/63	III	2	No	R-hyperCVAD	CR →Relapse	10.0	163.2	Alive, CR after salvage treatment (ESHAP 3 cycles → PD → DL-ICE 6 cycles → CR)
23	M/66	II bulky	1	No	R-CHOP →R-hyperCVAD	CR	11.3	11.3	Died, pneumonia in CR status
24	M/41	IV	1	Yes	R-CHOP →R-hyperCVAD	CR →Relapse	13.3	13.9	Died, hospice care without salvage

AlloHSCT, allogeneic stem cell transplantation; BM, bone marrow; CNS, cerebral nerve system; CR, complete remission; ECOG, Eastern Cooperative Oncology Group; EFS, event-free survival; OS, overall survival; PD, progressive disease; PR, partial remission; RSV, respiratory syncytial virus; TLS, tumor lysis syndrome; WBRT, whole brain radiotherapy.

The interim response evaluation revealed that 49 patients achieved CR (71.0%), 5 had PR (7.2%), 8 had PD (11.6%), and the remaining 7 died (10.1%) during the 1^st^ chemotherapy cycle (**Supplementary Figure S3**). All 49 patients who achieved CR maintained their remission status after completing chemotherapy; however, 3 of them relapsed, and 1 patient, who was still in CR, died from pneumonia (patient 23, **Table 2**). Among the three relapsed patients, two (patients 18 and 24, **Table 2**) died from PD without salvage treatment, while one (patient 22, **Table 2**) achieved CR after six DL-ICE salvage chemotherapy cycles, following PD after three ESHAP cycles. Among the five PR patients, one (patient 13, **Table 2**) died from fungal pneumonia in PR status, two (patients 14 and 19, **Table 2**) died from PD without salvage treatment, while two (patients 20 and 21, **Table 2**) achieved CR after salvage therapy. Patient 20 experienced systemic relapse with CNS involvement and underwent whole brain radiotherapy (WBRT), followed by four DL-ICE cycles and allo-HSCT. Patient 21 initially received three DL-ICE cycles but progressed, leading to four cycles of ESHAP and allo-HSCT. All patients developing PD (patients 8–12 and 15–17, **Table 2**) died shortly after due to disease progression.

During first-line treatment, all patients experienced at least one episode of hematologic toxicity of any grade, and most patients developed grade 3 or 4 events: neutropenia in 65 patients (94.2%), anemia in 36 (52.2%), and thrombocytopenia in 57 (82.6%). Patients treated with R-hyperCVAD/MC had a significantly higher incidence of grade 3 or 4 anemia (59.6% vs. 29.4%, p=0.030) and thrombocytopenia (88.5% vs. 64.7%, p=0.025) than those receiving mixed regimens of R-CHOP ± R-hyperCVAD/MC. No significant differences in non-hematologic adverse events were observed between groups during first-line treatment. In cycle 1 course A, 14 patients developed TLS, with 8 also experiencing concomitant acute kidney injury. Despite the implementation of all appropriate therapeutic measures, two patients succumbed to TLS, while the remaining recovered with intravenous hydration and rasburicase administration, without the need for hemodialysis. In total, 52 patients (75.4%) experienced infectious complications, and bacterial growth was confirmed in blood cultures from 43 patients (62.3%). Details of the adverse events during first-line treatment are summarized in **Supplementary Table S1**.

At diagnosis, nine patients (13.0%) had CNS involvement; of these eight were administered full-dose R-hyperCVAD/MC, while one (patient 24, **Table 2**) was switched from R-CHOP to R-hyperCVAD/MC. Among them, 7 achieved long-term CR, 1 (patient 15, **Table 2**) died from PD, and 2 relapsed (patient 14 with CNS parenchymal relapse and patient 24 with multifocal bone involvement, **Table 2**), both succumbing to PD. Additionally, two patients (patients 18 and 20, **Table 2**) experienced CNS relapse despite no initial CNS involvement. Patient 18 died without salvage treatment, while patient 20 achieved long-term CR through WBRT, DL-ICE salvage therapy, and allo-HSCT. Detailed clinical characteristics and long-term outcomes of treatment failure or patients with relapsed BL are summarized in **Table 2**.

### 3.3 Survival outcomes and prognostic factors

**Figure 1:**
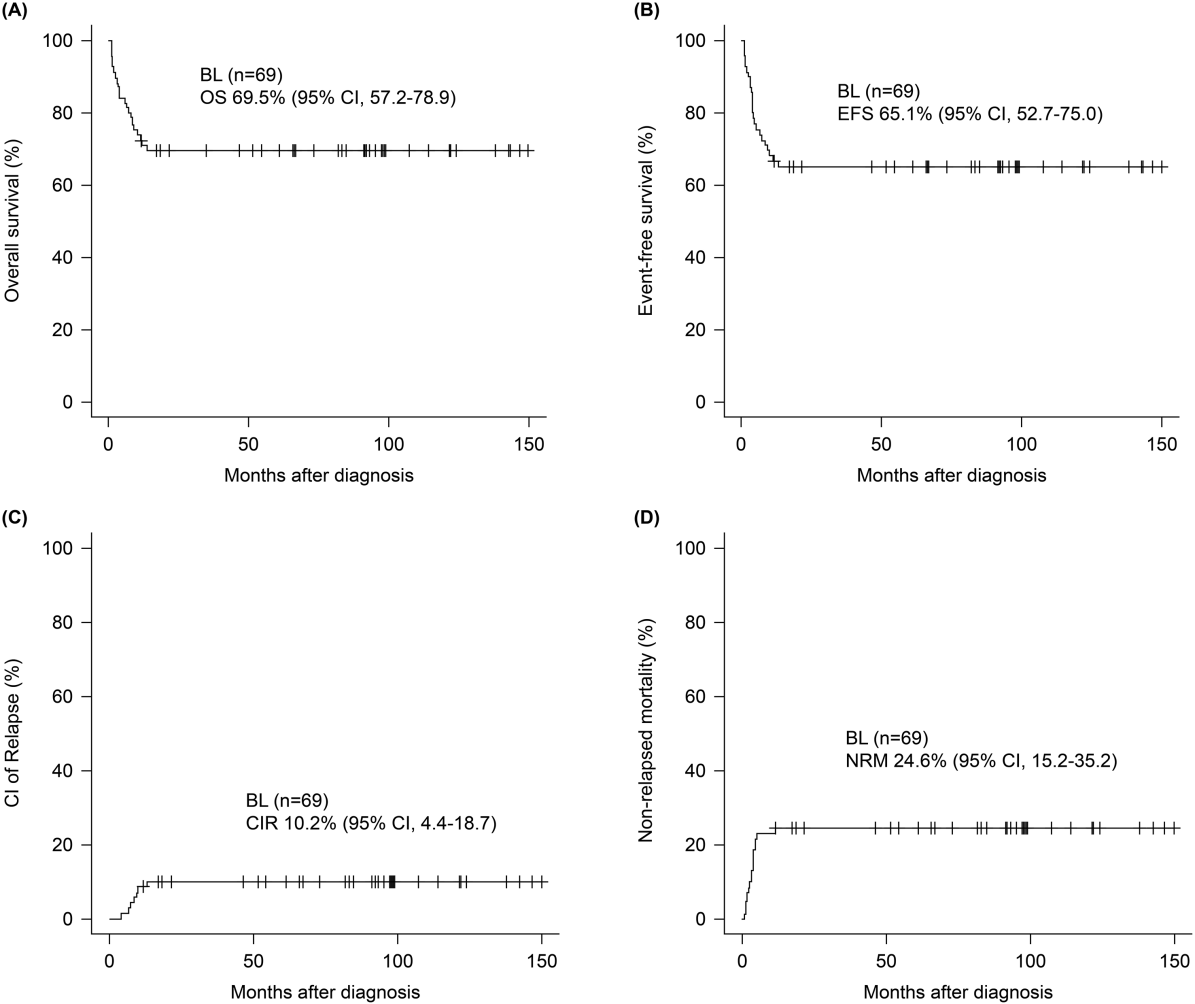
Survival outcomes of patients with Burkitt lymphoma. At a median follow-up of 66.9 months, 5-year **(A)** OS and **(B)** EFS were 69.5% (95% CI, 57.2–78.9) and 65.1% (95% CI, 52.7-75.0). The 5-year **(C)** CIR and **(D)** NRM were 10.2% (95% CI, 4.4–18.7) and 24.6% (95% CI, 15.2–35.2).

**Figure 2:**
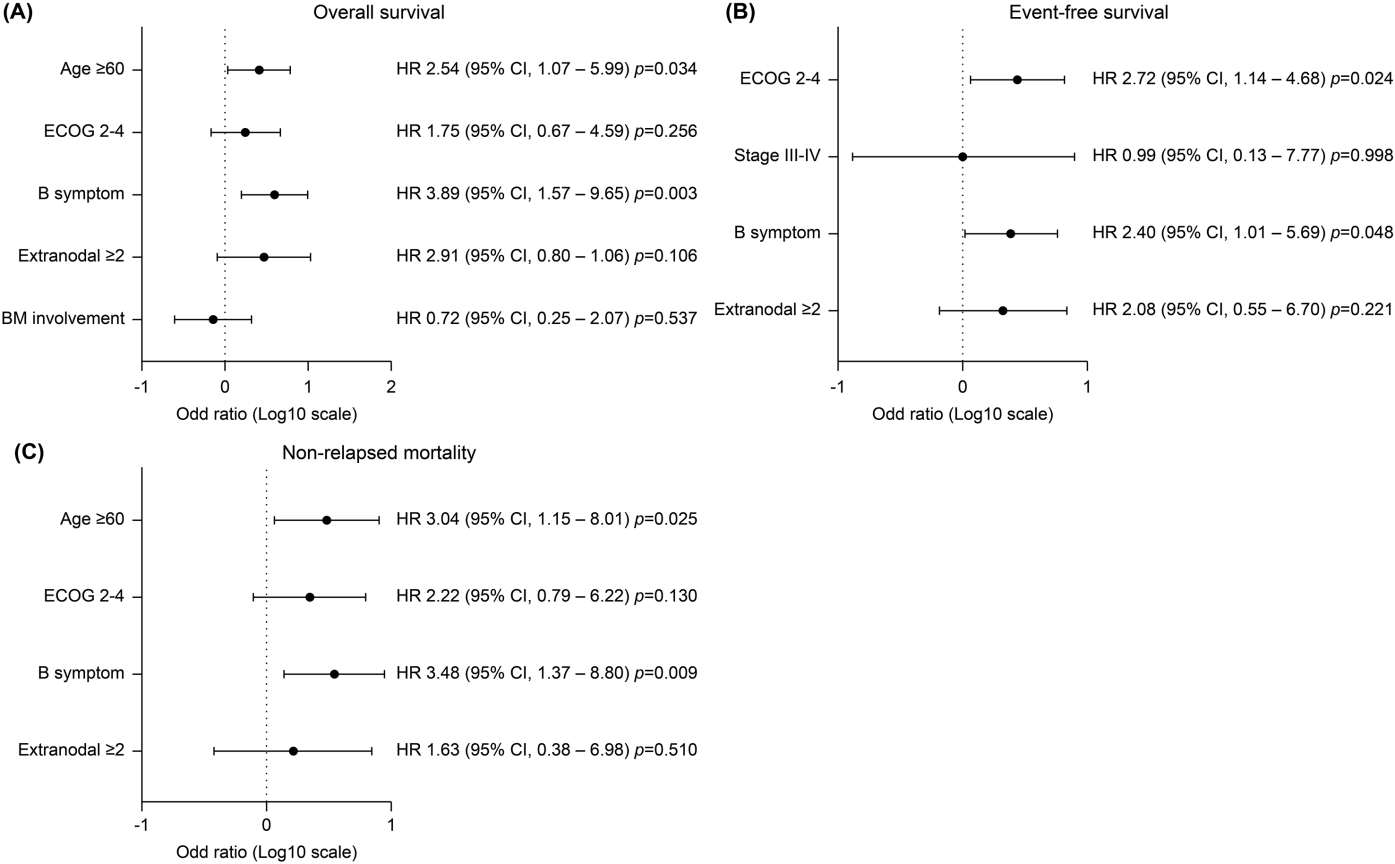
Multivariate analysis of progression free survival of significant covariates. **(A)** Age ≥ 60 years and the presence of B symptoms were significantly associated with inferior overall survival (HR 2.54, 95% CI 1.07–5.99, p=0.034; and HR 3.89, 95% CI 1.57–9.65, p=0.003, respectively). **(B)** Event-free survival was significantly worse in patients with poor performance status of ECOG 2–4 (HR 2.72, 95% CI 1.14–4.68, p=0.024) and in those with B symptoms (HR 2.40, 95% CI 1.01–5.69, p=0.048). **(C)** Non-relapse mortality was significantly higher in patients aged ≥60 years (HR 3.04, 95% CI 1.15–8.01, p=0.025) and those with B symptoms (HR 3.48, 95% CI 1.37–8.80, p=0.009).

The median follow-up period was 66.9 months (range 10.5–174.9) and the 5-year OS and EFS was 69.5 (95% confidence interval [CI], 57.2–78.9) and 65.1% (95% CI, 52.7-75.0), respectively (**Figures 1A, B**). The 5-year CIR and NRM was 10.2 (95% CI, 4.4–18.7) and 24.6% (95% CI, 15.2–35.2), respectively (**Figures 1C, D**). Survival outcomes stratified by initial treatment regimens—including patients treated with pure R-hyperCVAD/MC, R-CHOP, and those who switched regimens during treatment—are presented in **Supplementary Figure S4**. Although differences in survival outcomes were observed among these subgroups, they did not reach statistical significance. Associations between age, sex, ECOG, stage B-symptoms, extranodal involvement of ≥ 2, BM involvement, CNS involvement, and LDH elevation were evaluated by univariate analysis in each survival outcome to identify prognostic factors, presented in **Supplementary Table S2**. Multivariate analysis (**Figure 2**) revealed that the presence of B-symptoms was significantly associated with the OS (HR 3.89, 95% CI, 1.57–9.65, *p* = 0.003), EFS (HR 2.40, 95% CI, 1.01–5.69, *p* = 0.048), and NRM (HR 3.48, 95% CI, 1.37–8.80, *p* = 0.009). Age ≥ 60 years was significantly correlated with OS (HR 2.54, 95% CI, 1.07–5.99, *p* = 0.034) and NRM (HR 3.04, 95% CI, 1.15–8.01, *p* = 0.025), and ECOG-PS 2–4 was significantly correlated with EFS (HR 2.72, 95% CI, 1.14–4.68, *p* = 0.024).

## 4 Discussion

In this study, seven early deaths were observed during the first-line treatment due to severe infections (n = 5) including bacterial septic shock (n = 3), acute respiratory failure after RSV infection (n = 1), and invasive aspergillosis (n = 1) along with TLS-induced multiorgan failure (n = 2) before the availability of rasburicase at our institute. All patients that developed PD (n = 8) during the second or third cycle died from rapid BL progression. Among five PR cases at interim response evaluation, two died from PD, one from fungal pneumonia, and two achieved long-term CR after salvage chemotherapy. Among 49 patients who attained CR initially, 3 relapsed; of these, 2 died from PD, and 1 achieved long-term CR post-salvage therapy. One patient who attained CR died 5.3 months later from pneumonia septic shock. Overall, 21 deaths (30.4%) occurred from various factors including disease-related (n = 12), infection-related (n = 7), and TLS-associated (n = 2) causes with an initial CR rate of 71.0%. The median survival was 3.9 months (range, 1.0–12.3) with 66.7% of deaths occurring in patients older than the cohort’s median age of 55 years (range, 15–81), suggesting that older Asian adults were more vulnerable to treatment-related toxicity with most deaths occurring shortly after BL diagnosis (21). Moreover, the 5-year CR was 10.2%, while NRM was 24.6%, indicating that intensive chemotherapy-related toxicity and infectious complications were major contributors to treatment failure.

BL is highly responsive to intensive systemic chemotherapy owing to its rapid proliferation and aggressive nature with curative outcomes achievable through chemotherapy alone, regardless of its variant (22). However, the optimal first-line treatment strategy remains uncertain due to the limited randomized studies (23, 24). Most adult BL regimens are derived from pediatric treatment protocols, typically including cyclophosphamide, vincristine, methotrexate, doxorubicin, and cytarabine. These intensive, pediatric-based chemotherapy approaches have been reported to improve remission and survival rates in both children and adults (25). In the United States, a recent retrospective analysis across 30 centers identified CODOX-M/IVAC, hyperCVAD/MC, and DA-R-EPOCH as the most common BL regimens (6). Among these, DA-R-EPOCH has been proposed to be a preferable option for low-risk patients without BM or CNS involvement, owing to its association with fewer toxicities as compared to regimens with high-dose methotrexate and cytarabine (6). Furthermore, patients administered rituximab have been reported to have better survival outcomes (6). The DA-R-EPOCH regimen is designed to reduce dose intensity, making it suitable for patients with older age, frailty, or immunosuppression; however, it provides limited potential for CNS disease treatment (9, 26). Instead, the CODOX-M/IVAC regimen is often selected for fit and younger patients who are eligible for high-intensity therapy, as it includes CNS-penetrant chemotherapeutic agents such as high-dose methotrexate, providing adequate CNS coverage (10, 27).

However, no significant differences were observed in overall clinical outcomes among these treatment approaches. A European retrospective study comparing BL treatment regimens revealed no significant differences in survival outcomes between patients treated with different chemoregimens (28). Among 105 patients with BL treated with LMB, BFM, HOVON, or CODOX-M/IVAC, the 5-year PFS rate was 69%. Notably, CODOX-M/IVAC was the most effective regimen with respect to cost efficiency, treatment duration, and proportion of patients completing the full treatment regimen (28). For patients with BL and CNS involvement, CODOX-M/IVAC is the preferred regimen over DA-R-EPOCH, as a multicenter study indicated poorer outcomes with DA-R-EPOCH in these patients (9). However, a recently halted randomized trial identified no significant outcome differences between DA-R-EPOCH and R-CODOX-M/R-IVAC in patients with high-risk without CNS involvement, though the latter was associated with excessive toxicity and prolonged hospitalization (29). Therefore, older patients with low-risk BL and no CNS involvement may be treated with DA-R-EPOCH, while high-risk young and fit patients with CNS involvement should be administered CODOX-M/IVAC to reduce treatment-related mortality (9, 29).

Thomas DA et al. first reported the addition of rituximab to the hyper-CVAD regimen, demonstrating a CR rate of 86%, with 3 PR and 1 PD in 31 patients with BL or B-cell acute lymphoblastic leukemia (12). The 3-year OS, EFS, and disease-free survival were 89, 80, and 89%, respectively. Multivariate analysis identified age and rituximab addition as significant favorable factors. A long-term follow-up study on the R-hyperCVAD regimen for BL and high-grade B-cell lymphoma confirmed its efficacy in patients with high-risk and CNS relapse prevention (30). The 5-year OS, relapse-free survival, and CIR were 68, 86, and 0%, respectively, in patients < 60 years of age without CNS or BM involvement. Their cohort had a high frequency of baseline CNS (28%) and BM (73%) involvement, yet the 5-year CNS CIR was 6% (four CNS relapses), with baseline CNS involvement as a significant risk factor (*p* = 0.03) (30). This outcome is comparable to the DA-EPOCH-R regimen, associated with higher CNS relapse risk (CIR 13% vs. 2–4%) as compared to those with dose-intense regimens (31). In our cohort, CNS and BM involvement were observed in 9 (13.0%) and 26 (37.7%) patients, respectively, with 3 (4.3%) experiencing CNS relapse. While the CNS relapse rate in BL is reported to be approximately 10%, indicating poor prognosis (3, 6, 13), our data also suggest that R-hyperCVAD/MC with routine intrathecal treatment provides effective CNS disease control (9). Therefore, R-hyperCVAD/MC in Korean population had acceptable efficacy in patients with high-risk BL and CNS or BM involvement. However, as previously discussed, early mortality due to disease progression or infectious complications remains high at 24.6%, especially among older patients.

For risk factors associated with poor clinical outcomes, age ≥ 40 years, LDH > 3× upper normal limit, ECOG-PS ≥ 2, and CNS involvement are well established predictors of inferior survival (26). In our study, age > 60 years was significantly associated with poorer OS and NRM, aligning with previous reports (3, 6, 10, 12). Additionally, patients presenting with B-symptoms at diagnosis, indicative of high tumor burden and strong immune response, had significantly worse OS, EFS, and NRM. ECOG-PS 2–4 was also strongly correlated with poorer EFS. We believe that the poor clinical outcomes of BL are primarily determined by patient age and performance status at diagnosis, as well as the interim response to intensive chemotherapy. However, once remission is achieved, the relapse and treatment-related toxicity rate is relatively lower as compared to those with other high-grade lymphomas, including diffuse large B-cell lymphoma (32).

This study has some limitations. First, this study was a retrospective analysis conducted within a single healthcare system in South Korea, which may limit the generalizability of the findings. To date, no prospective studies have evaluated the efficacy of R-hyperCVAD/MC or compared it with alternative regimens such as CODOX-M/IVAC or DA-R-EPOCH in adult BL. Second, the relatively small sample size reduced the statistical power, further limiting the broader applicability of our results. The outcomes reported may reflect institution-specific and region-specific factors, including patient demographics, healthcare practices, and treatment accessibility. Nevertheless, given the rarity of adult BL in the Asian population, we believe that our findings offer meaningful insights into real-world treatment outcomes and prognosis in Korean patients. To address the above limitations, well-designed multicenter prospective studies are warranted to optimize treatment strategies, particularly for older or unfit patients, and to enable direct comparisons between R-hyperCVAD/MC and other intensive regimens. Further validation in diverse ethnic groups and healthcare systems is also essential to enhance external validity.

Some patients were administered R-CHOP instead of R-hyperCVAD/MC due to delayed diagnosis or challenges in administering intensive chemotherapy. While R-CHOP is suboptimal for BL treatment, a likely limitation of this study, it was used owing to the Korean health insurance policies that did not reimburse DA-R-EPOCH for BL and the clinical significance of rituximab-adriamycin combinations in BL treatment. Beyond the limitations of the Korean health insurance system, we believe DA-R-EPOCH should be reserved for patients with low-risk or those unable to tolerate more intensive regimens. Owing to the relatively high treatment-related toxicity and mortality of R-hyperCVAD/MC, future regimen modifications incorporating novel immunotherapies, including bispecific antibodies or chimeric antigen receptor T-cell therapy, may improve efficacy while minimizing toxicity.

In summary, the R-hyperCVAD/MC regimen is effective for Korean adult BL, resulting in a 5-year survival rate of 69.5%, particularly in patients with high-risk and BM or CNS involvement. However, its high-intensity leads to significant complications and early mortality, especially in patients aged > 60 years. R-CHOP is used as an alternative in cases with higher risk of treatment-related death, need for risk-adapted modifications or novel agent addition to improve outcomes.

## Data Availability

The datasets presented in this article are not readily available due to privacy concerns regarding patient data. Requests to access the datasets should be directed to Gi-June Min (beichest@nate.com).

## References

[1] Magrath I. The Pathogenesis of Burkitt’s Lymphoma. In: Vande Woude GF Klein G, editors. Advances in Cancer Research, vol. 55. Cambridge, Massachusetts, USA: Academic Press (1990). p. 133–270. 10.1016/s0065-230x(08)60470-4 2166998

[2] Bishop PC Rao VK Wilson WH. Burkitt’s Lymphoma: molecular pathogenesis and treatment. Can Investig. (2000) 18:574–83. doi: 10.3109/07357900009012197, PMID: 10923106

[3] Choi MK Jun HJ Lee SY Kim KH Lim DH Kim K. Treatment outcome of adult patients with Burkitt lymphoma: results using the LMB protocol in Korea. Ann Hematol. (2009) 88:1099–106. doi: 10.1007/s00277-009-0729-3, PMID: 19288103

[4] Kian TC Miriam T Richard Q Peng YS Li LE Ivy S. Clinical characteristics, prognostic factors and outcomes of Burkitt lymphoma in adult Asians. Leuk Lymph. (2008) 49:824–27. doi: 10.1080/10428190701882948, PMID: 18398754

[5] Park YH Kim WS Kang HJ Na II Ryoo B-Y Yang SH. Gastric Burkitt lymphoma is a distinct subtype that has superior outcomes to other types of Burkitt lymphoma/leukemia. Ann Hematol. (2006) 85:285–90. doi: 10.1007/s00277-005-0050-8, PMID: 16518604

[6] Evens AM Danilov A Jagadeesh D Sperling A Kim S-H Vaca R. Burkitt lymphoma in the modern era: real-world outcomes and prognostication across 30 US cancer centers. Blood. (2021) 137:374–86. doi: 10.1182/blood.2020006926, PMID: 32663292 PMC8765121

[7] Hoelzer D Walewski J Döhner H Viardot A Hiddemann W Spiekermann K. Improved outcome of adult Burkitt lymphoma/leukemia with rituximab and chemotherapy: report of a large prospective multicenter trial. Blood. (2014) 124:3870–79. doi: 10.1182/blood-2014-03-563627, PMID: 25359988 PMC4271177

[8] Dave SS Fu K Wright GW Lam LT Kluin P Boerma EJ. Molecular diagnosis of Burkitt’s lymphoma. NEJM. (2006) 354:2431–42. doi: 10.1056/NEJMoa055759, PMID: 16760443

[9] Roschewski M Dunleavy K Abramson JS Powell BL Link BK Patel P. Multicenter study of risk-adapted therapy with dose-adjusted EPOCH-R in adults with untreated Burkitt Lymphoma. J Clin Oncol. (2020) 38:2519–29. doi: 10.1200/jco.20.00303, PMID: 32453640 PMC7392744

[10] Barnes JA Lacasce AS Feng Y Toomey CE Neuberg D Michaelson JS. Evaluation of the addition of rituximab to CODOX-M/IVAC for Burkitt’s lymphoma: a retrospective analysis. Ann Oncol. (2011) 22:1859–64. doi: 10.1093/annonc/mdq677, PMID: 21339382

[11] Mead GM Barrans SL Qian W Walewski J Radford JA Wolf M. A prospective clinicopathologic study of dose-modified CODOX-M/IVAC in patients with sporadic Burkitt lymphoma defined using cytogenetic and immunophenotypic criteria (MRC/NCRI LY10 trial). Blood. (2008) 112:2248–60. doi: 10.1182/blood-2008-03-145128, PMID: 18612102 PMC2532802

[12] Thomas DA Faderl S O’Brien S Bueso-Ramos C Cortes J Garcia-Manero G. Chemoimmunotherapy with hyper-CVAD plus rituximab for the treatment of adult Burkitt and Burkitt-type lymphoma or acute lymphoblastic leukemia. Cancer. (2006) 106:1569–80. doi: 10.1002/cncr.21776, PMID: 16502413

[13] Diviné M Casassus P Koscielny S Bosq J Sebban C Le Maignan C. Burkitt lymphoma in adults: a prospective study of 72 patients treated with an adapted pediatric LMB protocol. Ann Oncol. (2005) 16:1928–35. doi: 10.1093/annonc/mdi403, PMID: 16284057

[14] Alaggio R Amador C Anagnostopoulos I Attygalle AD Araujo IBO Berti E. The 5th edition of the world health organization classification of haematolymphoid tumours: lymphoid neoplasms. Leukemia. (2022) 36:1720–48. doi: 10.1038/s41375-022-01620-2, PMID: 35732829 PMC9214472

[15] Jeon YW Kwak DH Park SS Yoon JH Lee SE Eom KS. Effectiveness of single-dose Rasburicase in patients with lymphoid Malignancies at a high risk for tumor lysis syndrome. Clin Lymph Myel Leuk. (2017) 17:595–603. doi: 10.1016/j.clml.2017.06.027, PMID: 28711571

[16] Min GJ Kim TY Jeon YW JH O BO C Park G. Diagnosis, treatment, and prognosis of primary intraocular lymphoma: Single-center real-world clinical experience. Cancer Med. (2023) 12:7911–22. doi: 10.1002/cam4.5567, PMID: 36721307 PMC10134376

[17] Cheson BD Fisher RI Barrington SF Cavalli F Schwartz LH Zucca E. Recommendations for initial evaluation, staging, and response assessment of Hodgkin and non-Hodgkin lymphoma: the Lugano classification. J Clin Oncol. (2014) 32:3059–68. doi: 10.1200/JCO.2013.54.8800, PMID: 25113753 PMC4979083

[18] Min GJ Jeon YW Park SS Shin SH Yahng SA Yoon JH. Poor prognosis in patients with diffuse large B cell lymphomas with bone marrow involvement possessing chromosomal abnormalities, despite aggressive treatment. Ann Hematol. (2020) 99:557–70. doi: 10.1007/s00277-020-03929-3, PMID: 31989249

[19] Malfona F Testi AM Chiaretti S Moleti ML. Refractory Burkitt Lymphoma: diagnosis and interventional strategies. Blood Lymph Cancer. (2024) 14:1–15. doi: 10.2147/blctt.S407804, PMID: 38510818 PMC10949171

[20] Jeon YW Yoon S Min GJ Park SS Park S Yoon JH. Clinical outcomes of Fludarabine and Melphalan with an 800 cGy total body irradiation conditioning regimen in patients with refractory or relapsed aggressive Non-Hodgkin Lymphoma undergoing allogeneic hematopoietic stem cell transplantation. Clin lymph Myel Leuk. (2019) 19:345–55.e347. doi: 10.1016/j.clml.2019.03.023, PMID: 31014757

[21] Kim K Johnson JA Derendorf H. Differences in drug pharmacokinetics between East Asians and Caucasians and the role of genetic polymorphisms. J Clin Pharmacol. (2004) 44:1083–105. doi: 10.1177/0091270004268128, PMID: 15342610

[22] McMaster ML Greer JP Greco FA Johnson DH Wolff SN Hainsworth JD. Effective treatment of small-noncleaved-cell lymphoma with high-intensity, brief-duration chemotherapy. J Clin Oncol. (1991) 9:941–6. doi: 10.1200/jco.1991.9.6.941, PMID: 1709685

[23] Jacobson C LaCasce A. How I treat Burkitt lymphoma in adults. Blood. (2014) 124:2913–20. doi: 10.1182/blood-2014-06-538504, PMID: 25258344

[24] Zayac AS Olszewski AJ. Burkitt lymphoma: bridging the gap between advances in molecular biology and therapy. Leuk Lymph. (2020) 61:1784–96. doi: 10.1080/10428194.2020.1747068, PMID: 32255708 PMC7429355

[25] Todeschini G Bonifacio M Tecchio C Balter R Carli G Stefani PM. Intensive short-term chemotherapy regimen induces high remission rate (over 90%) and event-free survival both in children and adult patients with advanced sporadic Burkitt lymphoma/leukemia. Am J Hematol. (2012) 87:22–5. doi: 10.1002/ajh.22189, PMID: 22086870

[26] Dunleavy K Pittaluga S Shovlin M Steinberg SM Cole D Grant C. Low-intensity therapy in adults with Burkitt’s lymphoma. N Engl J Med. (2013) 369:1915–25. doi: 10.1056/NEJMoa1308392, PMID: 24224624 PMC3901044

[27] Evens AM Carson KR Kolesar J Nabhan C Helenowski I Islam N. A multicenter phase II study incorporating high-dose rituximab and liposomal doxorubicin into the CODOX-M/IVAC regimen for untreated Burkitt’s lymphoma. Ann Oncol. (2013) 24:3076–81. doi: 10.1093/annonc/mdt414, PMID: 24146219 PMC3841019

[28] Oosten LEM Chamuleau MED Thielen FW de Wreede LC Siemes C Doorduijn JK. Treatment of sporadic Burkitt lymphoma in adults, a retrospective comparison of four treatment regimens. Ann Hematol. (2018) 97:255–66. doi: 10.1007/s00277-017-3167-7, PMID: 29209924 PMC5754407

[29] Chamuleau M Stenner F Chitu D Novak U Minnema M Geerts P. R-CODOX-M/R-IVA versus DA-EPOCH-R in patients with newly diagnosed Burkitt Lymphoma. final results of a multicenter randomized HOVON/SAKK trial. Lancet Haematol. (2023) 10:e966–75. doi: 10.1016/S2352-3026(23)00279-X, PMID: 37922925

[30] Samra B Khoury JD Morita K Ravandi F Richard-Carpentier G Short NJ. Long-term outcome of hyper-CVAD-R for Burkitt leukemia/lymphoma and high-grade B-cell lymphoma: focus on CNS relapse. Blood Adv. (2021) 5:3913–8. doi: 10.1182/bloodadvances.2021004427, PMID: 34464974 PMC8945626

[31] Zayac A Evens AM Stadnik A Smith SD Jagadeesh D Leslie LA. Outcomes of patients with newly-diagnosed Burkitt Lymphoma (BL) and central nervous system (CNS) involvement treated in the modern era: a multi-institutional real-world analysis. Blood. (2019) 134:402. doi: 10.1182/blood-2019-122990

[32] Crump M Neelapu SS Farooq U Van Den Neste E Kuruvilla J Westin J. Outcomes in refractory diffuse large B-cell lymphoma: results from the international SCHOLAR-1 study. Blood. (2017) 130:1800–8. doi: 10.1182/blood-2017-03-769620, PMID: 28774879 PMC5649550

