# Supplementary material for: Efficacy and safety of rituximab-based chemoimmunotherapy in adult patients with Burkitt lymphoma in Korea
Source: Front Oncol. 2025 Jul 9;15:1614506. doi: 10.3389/fonc.2025.1614506 (PMC12283665; doi:10.3389/fonc.2025.1614506)
Supplement: Supplementary file 1 [file DataSheet1.docx]

Supplementary Material

# Supplementary Figures and Tables

## Supplementary Figures

**
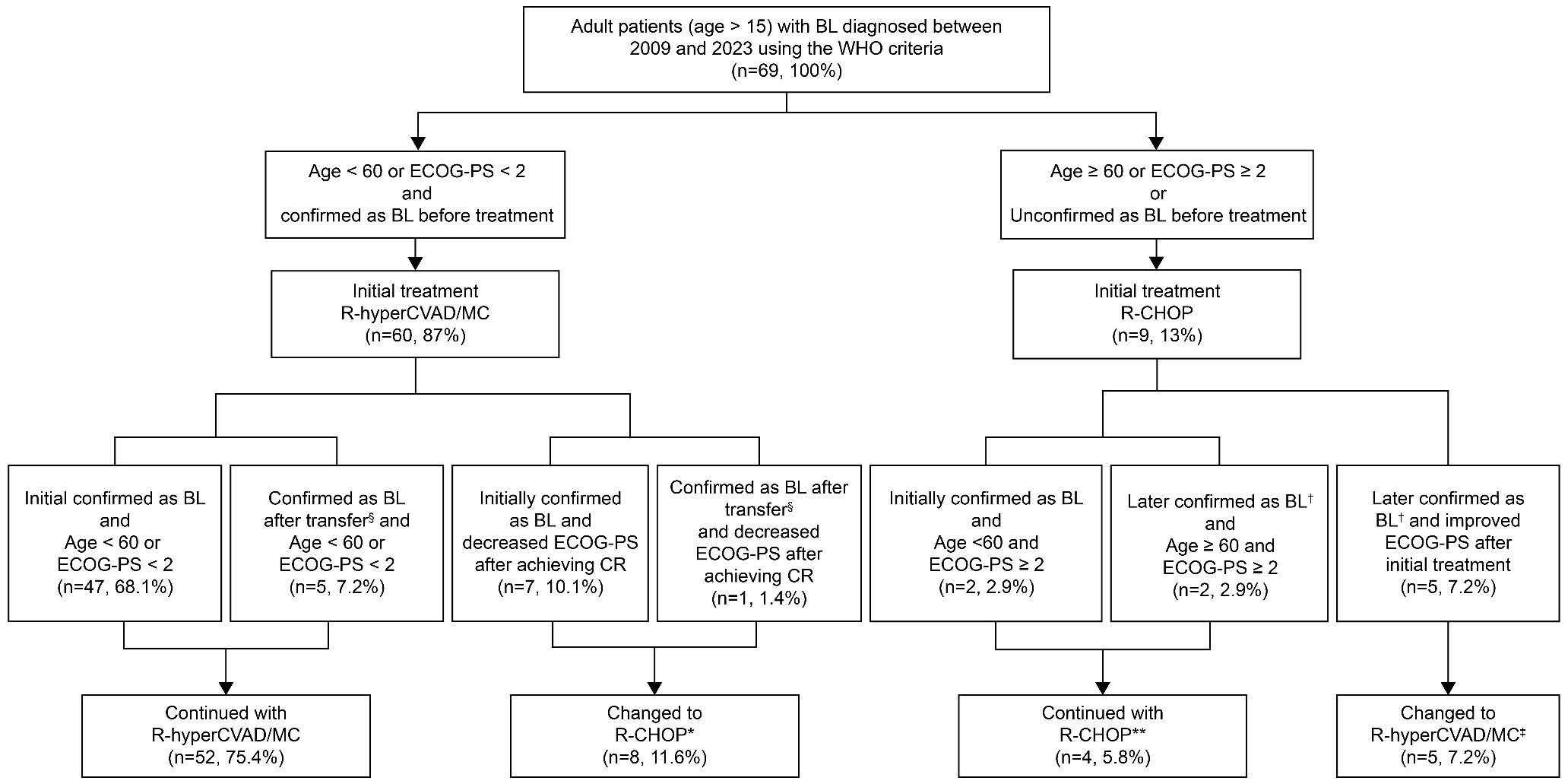
**

**Supplementary Figure S1. Flow diagram of treatment allocation and diagnostic confirmation in 69 adult patients with BL**

This flowchart illustrates treatment pathways for 69 adult patients (age >15 years) diagnosed with BL according to WHO criteria between 2009 and 2023. Patients were initially stratified by age and ECOG-PS, as well as diagnostic confirmation of BL. Most patients (n=60, 87%) received R-hyperCVAD/MC as initial treatment, whereas the remaining (n=9, 13%) received R-CHOP, primarily owing to older age, poor performance status, or delayed diagnostic confirmation. Among patients treated with R-hyperCVAD/MC, 52 (75.4%) continued the regimen throughout treatment, while 8 (11.6%) were switched to R-CHOP owing to decreased performance status after achieving CR. Five patients were initially diagnosed with high-grade B-cell lymphoma and later reclassified as BL after *MYC* testing; all were converted to R-hyperCVAD/MC.

§ These six patients were initially diagnosed with diffuse large B-cell lymphoma at other hospitals and were referred to our institution, where additional diagnostic work-up or pathological review led to a revised diagnosis of BL. Consequently, R-hyperCVAD/MC therapy could be promptly initiated.

† These seven patients were initially diagnosed with high-grade B-cell lymphoma and were later reclassified as BL after receiving 1–2 cycles of R-CHOP treatment.

* These patients underwent 2 cycles of R-hyperCVAD/MC and achieved CR but their performance status decreased making unable to continue R-hyperCVAD/MC. Thus, 2 more cycles of R-CHOP was administered to finish the treatment.

** These patients only received R-CHOP chemotherapy based on clinicians' assessment that their poor performance status and/or advanced age would make them unable to tolerate the intensity of R-hyperCVAD/MC.

‡ These patients received two cycles of R-CHOP, after which their treatment was switched to R-hyperCVAD/MC owing to a delayed diagnosis of BL. The physician assessed that the patients’ condition had improved sufficiently to tolerate the intensity of the R-hyperCVAD/MC regimen.

BL, Burkitt lymphoma; R-hyperCVAD/MC, rituximab, cyclophosphamide, doxorubicin, vincristine, and dexamethasone alternating with rituximab, methotrexate, and cytarabine; R-CHOP, rituximab, cyclophosphamide, doxorubicin, vincristine, and prednisone; CR, complete remission; ECOG, Eastern Cooperative Oncology Group; PS, performance status.


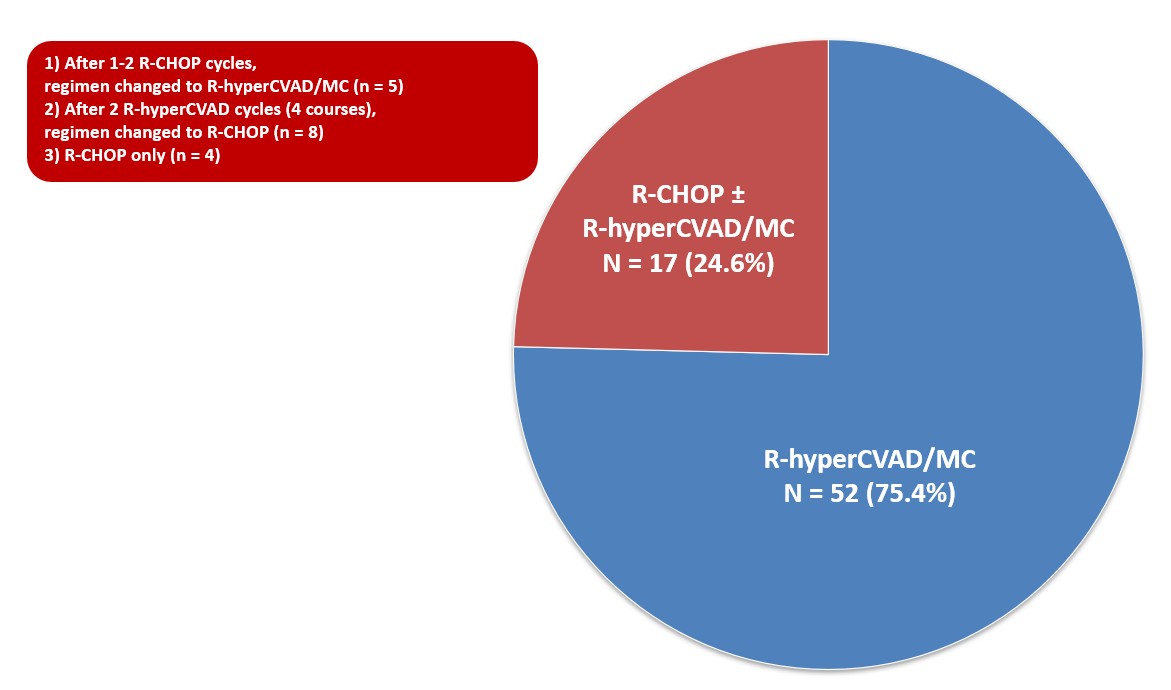


**Supplementary Figure S2.** Treatment modalities of enrolled patients with Burkitt lymphoma (n = 69). R-hyperCVAD/MC, rituximab, cyclophosphamide, doxorubicin, vincristine, and dexamethasone alternating with rituximab, methotrexate, and cytarabine; R-CHOP, rituximab, cyclophosphamide, doxorubicin, vincristine, and prednisone.


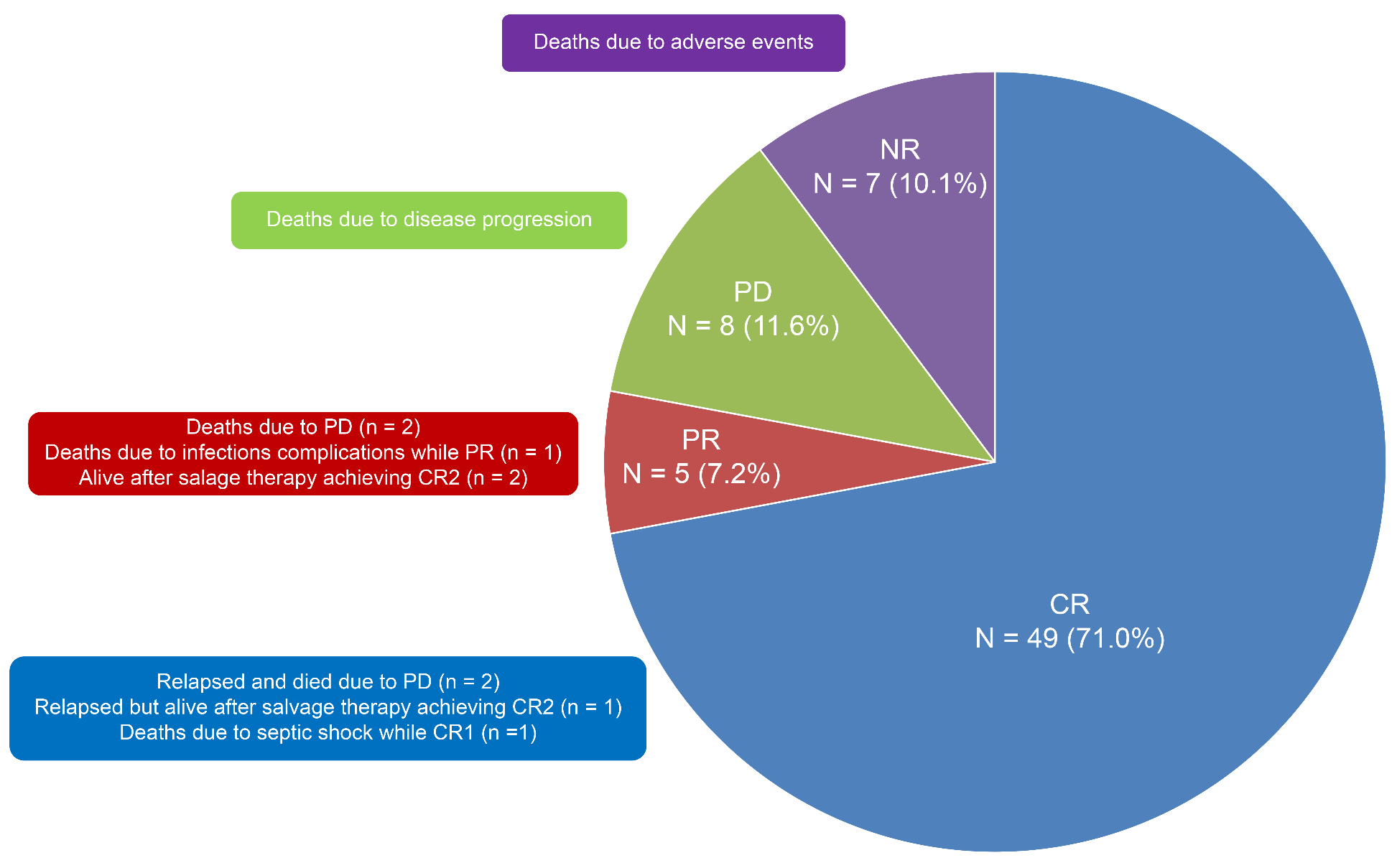


**Supplementary Figure S3.** Clinical outcomes of enrolled patients with Burkitt lymphoma (n = 69). R-hyperCVAD/MC, rituximab, cyclophosphamide, doxorubicin, vincristine, and dexamethasone alternating with rituximab, methotrexate, and cytarabine; R-CHOP, rituximab, cyclophosphamide, doxorubicin, vincristine, and prednisone.

**
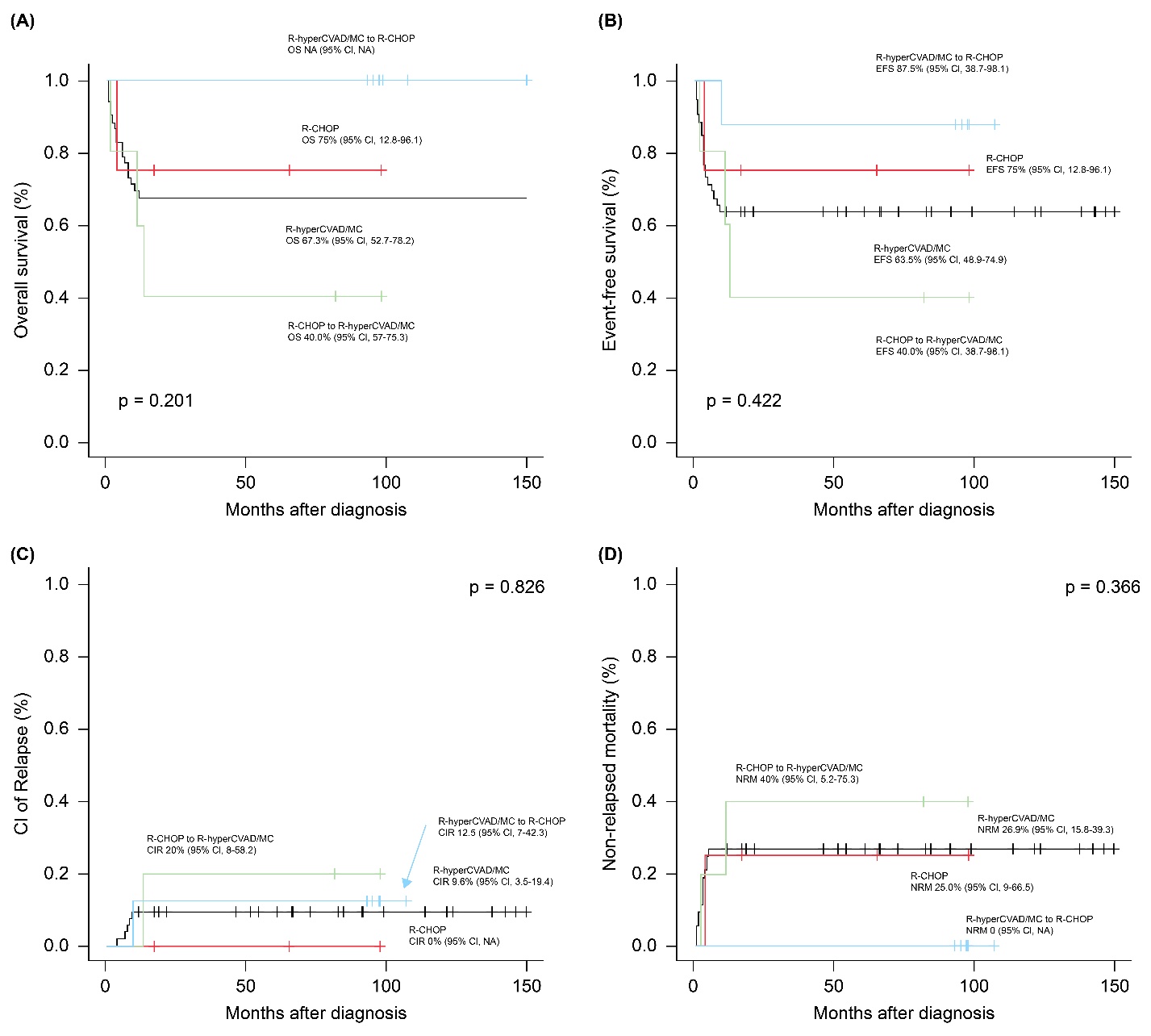
**

**Supplementary Figure S4.** Survival outcomes by subgroup. R-hyperCVAD/MC, rituximab, cyclophosphamide, doxorubicin, vincristine, and dexamethasone alternating with rituximab, methotrexate, and cytarabine; R-CHOP, rituximab, cyclophosphamide, doxorubicin, vincristine, and prednisone.

## Supplementary Tables

**Supplementary Table S1. Adverse Events During First-Line Therapy in Patients with BL (N = 69)**

| **Characteristics** | **R-hyperCVAD/MC only**  **(N=52)** | **R-CHOP±R-hyperCVAD/MC^*^**  **(N=17)** | **P-value** |
| --- | --- | --- | --- |
| **Hematologic** |  |  |  |
| ***NCI-CTC-AE Grade III-IV*^§^** |  |  |  |
| Neutropenia | 50 (96.2%) | 15 (88.2%) | 0.252 |
| Anemia | 31 (59.6%) | 5 (29.4%) | 0.030 |
| Thrombocytopenia | 46 (88.5%) | 11 (64.7%) | 0.025 |
| **Non-hematologic** |  |  |  |
| ***NCI-CTC-AE Any grades*** |  |  |  |
| Infection | 41 (78.8%) | 11 (64.7%) | 0.240 |
| Bacteremia, culture-proven | 35 (67.3%) | 8 (47.1%) | 0.135 |
| Peripheral neuropathy | 28 (53.8%) | 13 (76.5%) | 0.099 |
| Hepatopathy | 41 (78.8%) | 10 (58.8%) | 0.103 |
| Acute kidney injury | 26 (50.0%) | 9 (52.9%) | 0.833 |
| Tumor lysis syndrome | 13 (25.0%) | 1 (5.9%) | 0.089 |
| ***NCI-CTC-AE Grade III-IV*** |  |  |  |
| Infection | 23 (44.2%) | 7 (41.2%) | 0.825 |
| Bacteremia, culture-proven | 9 (17.3%) | 3 (17.6%) | 0.974 |
| Peripheral neuropathy | 19 (36.5%) | 9 (52.9%) | 0.232 |
| Hepatopathy | 12 (23.1%) | 4 (23.5%) | 0.969 |
| Acute kidney injury | 16 (30.8%) | 4 (23.5%) | 0.568 |
| Tumor lysis syndrome | 8 (15.4%) | 0 | 0.085 |

BL, Burkitt lymphoma; NCI-CTC-AE, national cancer institute common terminology criteria for adverse events version 5.0

^*^Patients in this group received either R-CHOP alone, R-CHOP followed by R-hyperCVAD/MC, or R-hyperCVAD/MC followed by R-CHOP.

^§^ All 69 patients experienced at least one episode of neutropenia, anemia, or thrombocytopenia of any grade during first-line treatment.

**Supplement Table S2. Univariate analysis of OS, PFS, CIR, and NRM in patients with BL**

| Variables | OS | *p-*value | EFS | *p-*value | CIR | *p-*value | NRM | *p-*value |
| --- | --- | --- | --- | --- | --- | --- | --- | --- |
| Age (years) |  | *0.045* |  | 0.056 |  | 0.722 |  | *0.026* |
| <60 (n = 45) | 77.8% (62.6-87.4) |  | 73.3% (57.8-83.9) |  | 11.1% (4.0-22.3) |  | 15.6% (6.8-27.7) |  |
| ≥60 (n = 24) | 53.8% (32.3-71.2) |  | 50.0% (29.1-67.8) |  | 8.3% (1.3-24.0) |  | 41.7% (21.7-60.5) |  |
| Sex |  | 0.054 |  | 0.059 |  | 0.290 |  | 0.154 |
| Male (n = 47) | 61.7% (46.3-73.9) |  | 57.4% (42.1-70.1) |  | 12.8% (5.1-24.1) |  | 29.8% (17.4-43.2) |  |
| Female (n = 22) | 86.4% (63.4-95.4) |  | 81.8% (58.5-92.8) |  | 4.5% (0.3-19.5) |  | 13.6% (3.3-31.3) |  |
| ECOG |  | *0.005* |  | *0.002* |  | 0.305 |  | *0.010* |
| 0-1 (n = 42) | 80.8% (65.3-89.9) |  | 78.5% (62.7-88.2) |  | 7.2% (1.8-17.8) |  | 14.3% (5.7-26.6) |  |
| ≥2 (n = 27) | 51.9% (31.9-68.5) |  | 44.4% (25.6-61.7) |  | 14.8% (4.4-31.1) |  | 40.7% (22.1-58.6) |  |
| Advance stage |  | 0.066 |  | *0.029* |  | 0.116 |  | 0.154 |
| Ann-Arbor stage I-II (n = 17) | 88.2% (60.6-96.9) |  | 88.2% (60.6-96.9) |  | 0% |  | 11.8% (1.8-31.9) |  |
| Ann-Arbor Stage III-IV (n = 52) | 63.5% (48.9-74.9) |  | 57.7% (43.2-69.7) |  | 13.5% (5.8-24.3) |  | 28.8% (17.2-41.5) |  |
| B symptoms |  | *0.002* |  | *0.003* |  | 0.361 |  | *0.014* |
| No (n = 41) | 82.9% (67.5-91.5) |  | 78.0% (62.1-87.9) |  | 7.3% (1.8-18.0) |  | 14.6% (5.9-27.2) |  |
| Yes (n = 28) | 50.0% (30.6-66.6) |  | 46.4% (27.6-63.3) |  | 14.3% (4.3-30.1) |  | 39.3% (21.3-56.9) |  |
| Extranodal involvement ≥2 |  | *0.010* |  | *0.009* |  | 0.177 |  | *0.048* |
| No (n = 26) | 88.5% (68.4-96.1) |  | 84.6% (64.0-93.9) |  | 3.8% (0.3-16.8) |  | 11.5% (2.8-27.1) |  |
| Yes (n = 43) | 58.1% (42.1-71.2) |  | 53.5% (37.6-67.0) |  | 14.0% (5.5-26.1) |  | 32.6% (19.1-46.7) |  |
| Bone marrow involvement |  | *0.039* |  | 0.119 |  | 0.718 |  | 0.178 |
| No (n = 43) | 79.0% (63.5-88.5) |  | 72.0% (56.0-83.0) |  | 9.4% (2.9-20.5) |  | 18.6% (8.6-31.5) |  |
| Yes (n = 26) | 53.8% (33.3-70.6) |  | 53.8% (33.3-70.6) |  | 11.5% (2.8-27.3) |  | 34.6% (17.1-52.9) |  |
| CNS involvement |  | 0.994 |  | 0.799 |  | 0.204 |  | 0.295 |
| No (n = 60) | 70.0% (56.6-79.9) |  | 65.0% (51.5-75.6) |  | 8.3% (3.0-17.1) |  | 26.7% (16.2-38.3) |  |
| Yes (n = 9) | 66.7% (28.2-87.8) |  | 66.7% (28.2-87.8) |  | 22.2% (2.8-53.3) |  | 11.1% (0.5-40.9) |  |
| Lactate dehydrogenase >3 x UNL |  | 0.152 |  | 0.202 |  | 0.416 |  | 0.420 |
| No (n = 39) | 76.8% (60.2-87.2) |  | 71.7% (54.7-83.2) |  | 7.8% (2.0-19.1) |  | 20.5% (9.5-34.4) |  |
| Yes (n = 30) | 60.0% (40.5-75.0) |  | 56.7% (37.3-72.1) |  | 13.3% (4.1-28.2) |  | 30.0% (14.8-46.9) |  |
| IPI score |  | *0.001* |  | *0.002* |  | 0.810 |  | *0.003* |
| 0-3 (n = 42) | 83.3% (68.1-91.7) |  | 78.5% (62.7-88.2) |  | 9.6% (3.0-20.9) |  | 11.9% (4.3-23.7) |  |
| 4-5 (n = 27) | 48.1% (28.7-65.2) |  | 44.4% (25.6-61.7) |  | 11.1% (2.6-26.5) |  | 44.4% (25.1-62.2) |  |

BL, Burkitt lymphoma; CIR, cumulative incidence of relapse; EFS, event-free survival; NRM, non-relapsed mortality; OS, overall survival

† Univariate analysis variables were selected based on prior literature on known prognostic factors.
